# Urban health: an example of a “health in all policies” approach in the context of SDGs implementation

**DOI:** 10.1186/s12992-019-0529-z

**Published:** 2019-12-18

**Authors:** Oriana Ramirez-Rubio, Carolyn Daher, Gonzalo Fanjul, Mireia Gascon, Natalie Mueller, Leire Pajín, Antoni Plasencia, David Rojas-Rueda, Meelan Thondoo, Mark J. Nieuwenhuijsen

**Affiliations:** 1 Barcelona Institute for Global Health, ISGlobal, Barcelona, Spain; 2 Universitat Pompeu Fabra (UPF), Barcelona, Spain; 3 CIBER Epidemiología y Salud Pública (CIBERESP), Barcelona, Spain; 4 Hospital Clínic-Universitat de Barcelona (UB), Barcelona, Spain; 5 Environmental and Radiological Health Sciences, Colorado State University, Fort Collins, USA; 6 University of Amsterdam, AISSR, Amsterdam, The Netherlands

**Keywords:** Sustainable development goals, Urban health, City planning, Transportation, Environmental health, Health equity, Health in all policies, Health promotion, Policy making, Health impact assessments

## Abstract

**Background:**

Cities are an important driving force to implement the Sustainable Development Goals (SDGs) and the New Urban Agenda. The SDGs provide an operational framework to consider urbanization globally, while providing local mechanisms for action and careful attention to closing the gaps in the distribution of health gains. While health and well-being are explicitly addressed in SDG 3, health is also present as a pre condition of SDG 11, that aims at inclusive, safe, resilient and sustainable cities.

Health in All Policies (HiAP) is an approach to public policy across sectors that systematically takes into account the health implications of decisions, seeks synergies, and avoids harmful health impacts in order to improve population health and health equity. HiAP is key for local decision-making processes in the context of urban policies to promote public health interventions aimed at achieving SDG targets. HiAPs relies heavily on the use of scientific evidence and evaluation tools, such as health impact assessments (HIAs). HIAs may include city-level quantitative burden of disease, health economic assessments, and citizen and other stakeholders’ involvement to inform the integration of health recommendations in urban policies.

The Barcelona Institute for Global Health (ISGlobal)‘s Urban Planning, Environment and Health Initiative provides an example of a successful model of translating scientific evidence into policy and practice with regards to sustainable and healthy urban development. The experiences collected through ISGlobal’s participation implementing HIAs in several cities worldwide as a way to promote HiAP are the basis for this analysis.

**Aim:**

The aim of this article is threefold: to understand the links between social determinants of health, environmental exposures, behaviour, health outcomes and urban policies within the SDGs, following a HiAP rationale; to review and analyze the key elements of a HiAP approach as an accelerator of the SDGs in the context of urban and transport planning; and to describe lessons learnt from practical implementation of HIAs in cities across Europe, Africa and Latin-America.

**Methods:**

We create a comprehensive, urban health related SDGs conceptual framework, by linking already described urban health dimensions to existing SDGs, targets and indicators. We discuss, taking into account the necessary conditions and steps to conduct HiAP, the main barriers and opportunities within the SDGs framework. We conclude by reviewing HIAs in a number of cities worldwide (based on the experiences collected by co-authors of this publication), including city-level quantitative burden of disease and health economic assessments, as practical tools to inform the integration of health recommendations in urban policies.

**Results:**

A conceptual framework linking SDGs and urban and transportplanning, environmental exposures, behaviour and health outcomes, following a HiAP rationale, is designed. We found at least 38 SDG targets relevant to urban health, corresponding to 15 SDGs, while 4 important aspects contained in our proposed framework were not present in the SDGs (physical activity, noise, quality of life or social capital). Thus, a more comprehensive HiAP vision within the SDGs could be beneficial.

Our analysis confirmed that the SDGs framework provides an opportunity to formulate and implement policies with a HiAP approach. Three important aspects are highlighted: 1) the importance of the intersectoral work and health equity as a cross-cutting issue in sustainable development endeavors; 2) policy coherence, health governance, and stakeholders’ participation as key issues; and 3) the need for high quality data.

HIAs are a practical tool to implement HiAP. Opportunities and barriers related to the political, legal and health governance context, the capacity to inform policies in other sectors, the involvement of different stakeholders, and the availability of quality data are discussed based on our experience. Quantitative assessments can provide powerful data such as: estimates of annual preventable morbidity and disability-adjusted life-years (DALYs) under compliance with international exposure recommendations for physical activity, exposure to air pollution, noise, heat, and access to green spaces; the associated economic impacts in health care costs per year; and the number of preventable premature deaths when improvements in urban and transport planning are implemented. This information has been used to support the design of policies that promote cycling, walking, public, zero and low-emitting modes of transport, and the provision of urban greening or healthy public open spaces in Barcelona (e.g. Urban Mobility, Green Infrastructure and Biodiversity Plans, or the Superblocks’s model), the Bus Rapid Transit and Open Streets initiatives in several Latin American cities or targeted SDGs assessments in Morocco.

**Conclusions:**

By applying tools such as HIA, HiAP can be implemented to inform and improve transport and urban planning to achieve the 2030 SDG Agenda. Such a framework could be potentially used in cities worldwide, including those of less developed regions or countries. Data availability, taking into account equity issues, strenghtening the communication between experts, decision makers and citizens, and the involvement of all major stakeholders are crucial elements for the HiAP approach to translate knowledge into SDG implementation.

## Background

The unprecedented changes over the past decades have led to an increase in complexity of social structures, global health problems and inequities within and across nations. Climate change challenges and the epidemiological and demographic transitions leading to rising non-communicable diseases and aging populations require reshaping how we develop public policies towards health [1]. Cities are home to more than half of the world’s population [2], and the urban context offers an unprecedented opportunity to understand the linkages between health, its social determinants and the environment, and to implement solutions following an intersectoral approach.

A deeper understanding of the inter-linkages in the way cities are designed, planned, built and governed and how this directly affects human health has evolved significantly in recent years. Two global milestones have pushed the idea that local decision-making processes that recognize urban policies are, in fact, key public health interventions. The first is the approval in 2015 of the 2030 Sustainable Development Agenda [3], comprised of 17 Sustainable Development Goals (SDGs) and 169 targets, with a global geographical scope. The SDGs provide, for the first time, an operational framework that tacitly calls for considering urbanization globally, while providing local mechanisms for action and careful attention to closing gaps in the distribution of health gains. The second milestone occurred in 2016, with the newly adopted New Urban Agenda at Habitat III, the United Nations Conference on Housing and Sustainable Urban Development [2]. This was the first time that ‘health’ appeared as a cross-cutting issue, and was explicitly acknowledged as a central component of urban planning and governance, beyond the provision of health care services. The WHO reinforced these links by gathering the increasing scientific evidence that connects the quality of urban design and transport with a variety of health outcomes [4].

However, these linkages are still not fully integrated into policy implementation. We propose that the paradigm of “Health in All Policies” (HiAP), and specific implementation tools (e.g. Health Impact Assessments, HIAs), could further advance the SDGs related to urban health. The aim of this article is threefold, first, to construct a conceptual framework that links social determinants of health, environmental exposures, behaviour and health outcomes with urban policies contained in the 2030 Sustainable Development Agenda and beyond, following a HiAP rationale. Second, to review and analyze the key elements of a HiAP approach in the context of urban and transport planning, paying particular attention to opportunities for advancing SDGs implementation. Third, to examine different examples of HIA in cities worldwide to understand the barriers and opportunities of this tool to support practical implementation of HiAP.

## Methods

We selected the conceptual urban health framework proposed by Nieuwenhuijsen in 2016 [5] that outlines the links between urban planning, behaviors, environmental exposures, and health outcomes that are key for urban and transport policies. We expanded the original framework to include other social determinants of health and health outcomes relevant to urban health as per Dahlgren and Whitehead [6]. We then linked each of those dimensions (i.e. health outcomes, urban health determinants or urban interventions) to one of the 17 SDGs or, when possible, to specific SDG’s targets and indicators. Evidence linking each component of the framework to urban health is briefly presented.

Taking into account this comprehensive view of “urban health”, we describe the HiAP approach, its necessary preconditions and its main components. Based on a previous general analysis by Ramirez et al. (2018) [7], comparing the interlinkages between HiAP and the SDGs, we review the opportunities presented within the SDGs framework to further advance HiAP and viceversa, this time tailored to the urban planning and transport sector policies.

Lastly, we focus on HIAs as a practical tool that promotes HiAP implementation by using scientific evidence and evaluation to inform the integration of health recommendations in the context of urban policies. We use examples of HIAs conducted by researchers of the Barcelona Institute for Global Health (ISGlobal)‘s Urban Planning, Environment and Health Initiative in more than 20 cities from low to high-income countries. Finally, we discuss the main barriers and bottlenecks, but also opportunities to achieve the SDGs created through these “in field” processes.

## Results

### Urban health within the SDGs: a conceptual framework

**Fig. 1:**
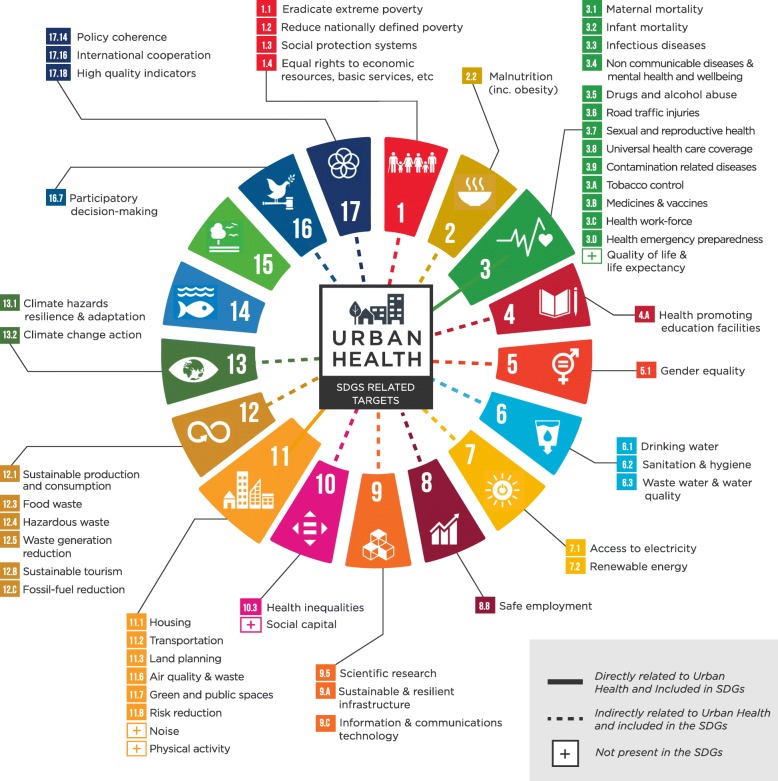
Conceptual Framework: Urban Health related SDGs within a HiAP approach

Based on our analysis using the Nieuwenhuijsen urban environmental health framework [5], a more comprehensive Urban Health Framework explicitly linked to the SDGs is presented in Fig. 1. At least 48 SDG targets have been included, corresponding to 15 SDGs (see

Table 1 for the original wording of related goals, targets and indicators). Four issues contained in this framework are not present in the SDGs (indicated by a “+” symbol). The selected urban health related SDG targets maintain their original colors used by the SDGs and constitute a mixture of health outcomes, environmental exposures and social determinants of health.

Table 1. Urban health related sustainable development goals, targets and indicators.

To further conceptualize these elements, the strength of the relationship among them is indicated by a continuous line (directly related to urban health and within the SDGs) and a dotted line (indirect relationship). The core of the figure is “urban health” as an intersectoral arena that links both the public health and the urban planning sectors, mainly captured by SDG 3 (Health and wellbeing) and SDG11 (inclusive, safe, resilient and sustainable cities), which appear bigger than the rest. 14 SDG3 related targets were included such as morbidity and mortality by infectious diseases (e.g. mosquito-borne or water-borne infections) that highly impact child mortality in developing countries; premature deaths due to non-communicable diseases such as cardiovascular illnesses or cancer, and those diseases directly linked to exposure to hazardous chemicals and air, water and soil pollution and contamination. There is a specific SDG target on deaths and injuries from road traffic accidents, highly relevant for city-based transportation policies, to be halved by 2030. Mental health and wellbeing promotion are also captured in the SDGs, as well as drugs and alcohol abuse and tobacco regulations. Most of these health outcomes are measured in terms of mortality, and, when available, incidence and prevalence (see indicators in (Table 1). However, other important dimensions, like quality of life measured by more sophisticated indicators such as life expectancy or self-perceived health, are currently not included in SDG3 UN proposed indicators (see the + mark). Other targets are more centered on health care systems and services such as universal health coverage, vaccines, health work force or emergency preparedness, that also need to be dealt at the city-level.

SDG 11 targets pertain to detrimental elements such as air pollution, but also positive environmental exposures, like green open spaces. Others, such as noise pollution, are not currently present in the SDGs framework. Physical activity, a key healthy lifestyle behavior, is also not present in any SDG target or indicator but “sports” are recognized as an SDG enabler in point 37 of the 2030 Agenda UN resolution (A/RES/70/1 Transforming our world: the 2030 Agenda for Sustainable Development, UN 2015). SDG 11.2 relates to “access to safe, affordable, accessible and sustainable transport systems for all”, notably by expanding public transport but also, although not explicitly mentioned in the SDGs, by promoting cycling and walking.

Other less directly related sectors that affect urban health include water and sanitation (SDG 6), access to quality education (SDG 4), decent and safe employment (SDG 8), and a nutritious diet (SDG 2) - with the goal of ending malnutrition, both from stunting and wasting, but also from overweight and obesity. SDG 13 contains both goals related to exposure to higher temperatures and other climate effects, but also actions aimed at “strengthening resilience and adaptive capacity to climate-related hazards and natural disasters”, particularly in human settlements such as cities. Also indirectly related are key aspects of electricity access and non-polluting energy sources (SDG 7), sustainable production and consumption patterns (SDG 12) or sustainable and resilient infrastructure, innovation and research (SDG 9).

Social determinants of health such as gender and socio-economic inequalities are captured by SDGs1, 5 and 10, but also in concrete targets and indicators within other SDGs that put the focus on the needs of those in vulnerable situations, women, children, people with disabilities and the elderly. Finally, social capital, a dimension that encompasses social connections and networks that catalyze cooperation and enable better (social and/or economic) outcomes [8], is related to health, but is not cited explicitly within the SDGs. Nevertheless, conditions for social capital and cohesion to happen such as peace, justice, and participatory processes, are contained in several targets of SDGs 16 and 17 and within other SDGs. The only two SDGs not included in this conceptual framework relate to marine, terrestrial and inland freshwater resources and ecosystems (SDGs 14 and 15) such as protected oceans, forests, wetlands, mountains and drylands. While city design and resource management affects land use, water reservoirs and other ecosystem elements, capturing these effects was outside the scope of this framework at this time.

All of these urban health-related SDGs and targets are interconnected. For example, there is enough scientific evidence to link lifestyle and dietary habits with health outcomes like obesity and diabetes [9], air pollution with cardiovascular, respiratory diseases and cancers [10] or noise pollution with mental health problems and cardiovascular diseases [11]. These elements are also dependent of each other and, thus, susceptible to changes as a consequence of urban planning and transport policies and interventions. Indeed, a growing body of scientific evidence on the health impacts of urban policies can clarify risks and inform decision-making for sustainable development [12]. Healthy urban policies can significantly reduce infectious and non-communicable diseases and enhance wellbeing. For example, compact urban design capitalizes on population density to reduce greenhouse gas emissions and improve mobility, walkability and social cohesion, and thereby health and well-being [13]. Efficient public transport in combination with cycling networks promote more physical activity, decrease air pollution, and reduce overall traffic deaths and injuries [14]. Green and blue open spaces in and around cities (e.g. green belts or urban riversides) improve resilience to heat waves, prevent city residents from heat island effect, provide corridors of less polluted air, enhance biodiversity and promote physical exercise [15]. Preservation of watersheds reduces drinking water contamination, saving on the costs of water purification. Recycling, reusing and reducing solid waste eliminates the need to burn or bury it, improving air quality, reducing water and soil contamination. Better wastewater and sewage management, in a context of rising temperatures and extreme weather events related to climate change, also improves public health by reducing exposure to water and mosquito-borne illnesses, such as recent urban epidemics of Zika or Chikungunya [4]. Taken together, these exposures contribute to the preventable burden of disease due to cardiovascular, respiratory, mental health, infectious diseases, and cancer morbidities, as well as an overall premature mortality. Thus, urban policies can significantly contribute to avoid premature deaths and provide cost-savings for the health care systems [16].

### Opportunities for a HiAP approach in the SDGs’ framework

The World Health Organization (WHO) defines HiAP as “*an approach to public policies across sectors that systematically takes into account the health implications of decisions, seeks synergies, and avoids harmful health impacts in order to improve population health and health equity*” [17]. The HiAP strategy provides a strong and effective “horizontal governance” [18] approach to complex health problems that involves the highest levels of government, political and executive leadership leading to effective priority setting, innovation in policy making and implementation of sustainable solutions.

Table 1 shows the necessary conditions to implement a HiAP at a national or local level as proposed by Leppo and colleagues [19]. The primary condition necessary is a supportive political and legal context. Capacity building, resources and quality data can be built upon this initial supportive context.

**Table 2:** Pre-existing conditions and components of HiAP

Pre-existing conditions necessary to conduct HiAP	Components of HiAP
1. Supportive context with: • political will • legal backing • governance structures and processes for intersectoral communication and implementation 2. Resources and skills to: • analyze impacts of major policies and policy proposals from the health perspective • communicate and negotiate across sectors • implement policy decisions • follow up policies’ impacts on determinants of health, and their distribution 3. Information on: • health situation and causes of ill-health, including distributional data on health inequities • potential health threats and exposures • effective policies/interventions from the health perspective, policy trends and proposals being developed across sectors, policy processes and actors beyond the health sector involved Source: extracted from Ministry of Social Affairs and Health, Finland, 2013 [19]	1. Establish the needs and priorities for HiAP; 2. Frame planned action; 3. Identify supportive structures and processes; 4. Facilitate assessment and engagement; 5. Ensure monitoring, evaluation and reporting; 6. Build capacity. Examples of HiAP indicators include participation of actors (by type, sectors or level), changes in organizational structures and culture (e.g. interministerial or inter-departmental committees), opportunities for joint actions, and willingness to share information and expertise. Source: HiAP by WHO’s HiAP Framework for Country Action [20]

Table 2 also captures the proposed components of WHO’s HiAP Framework for Country Action [20].

A previous analysis by Ramirez et al. [7] highlighted how HiAP and the SDGs are complementary approaches to consider sustainable development systemically. One of the main conclusions is that the SDGs framework provides an opportunity to formulate and implement policies with a HiAP approach. Below we further detail several elements of the SDGs framework that stand out as highly relevant to further advance HiAP, particularly for urban contexts.

#### 1) The SDGs provide a platform for intersectoral work

Contributions from sectors other than health (see Table 2) are instrumental to achieve progress towards healthy lives and well-being for all (SDG3). In addition, for the first time, the more traditional health related goals from the previous development agenda (e.g. maternal and child health, and infectious diseases), are coupled with non-communicable and mental diseases and environmental and socioeconomic determinants to provide a truly comprehensive picture of global health challenges and define responses accordingly. Within this context of rising NCDs as global health and sustainable development priorities, HiAP has also gained prominence. As stated in the UN political declaration on the prevention and control of NCDs, whole-of-government and whole-of-society approaches (i.e. HiAP) are needed to prevent and slow down current epidemics of chronic diseases and their main risk factors [21].

**Table 1:** Urban health related sustainable development goals, targets and indicators by HiAP key aspects

Sustainable Development Goals and targets related to Urban Health	Indicators proposed by the UN Statistical Commission (2016)
***Intersectoral approach: key SDGs related to Urban Health***	
**SDG 3: Ensure healthy lives and promote well-being for all**	
3.1 By 2030, reduce the global maternal mortality ratio to less than 70 per 100,000 live births	3.1.1 Maternal mortality ratio
3.2 By 2030, end preventable deaths of newborns and children under 5 years of age, with all countries aiming to reduce neonatal mortality to at least as low as 12 per 1000 live births and under-5 mortality to at least as low as 25 per 1000 live births	3.2.1 Under-five mortality rate
3.3 By 2030, end the epidemics of AIDS, tuberculosis, malaria and neglected tropical diseases and combat hepatitis, water-borne diseases and other communicable diseases	3.3.3 Malaria incidence per 1000 population 3.3.5 Number of people requiring interventions against neglected tropical diseases
3.4 By 2030, reduce by one third premature mortality from non-communicable diseases through prevention and treatment and promote mental health and well-being	3.4.1 Mortality rate attributed to cardiovascular disease, cancer, diabetes or chronic respiratory disease 3.4.2 Suicide mortality rate
3.5 Strengthen the prevention and treatment of substance abuse, including narcotic drug abuse and harmful use of alcoho	3.5.2 Harmful use of alcohol, defined according to the national context as alcohol per capita consumption (aged 15 years and older) within a calendar year in litres of pure alcohol
3.6 By 2020, halve the number of global deaths and injuries from road traffic accidents	3.6.1 Death rate due to road traffic injuries
3.7 By 2030, ensure universal access to sexual and reproductive health-care services, including for family planning, information and education, and the integration of reproductive health into national strategies and programmes	3.7.1 Proportion of women of reproductive age (aged 15–49 years) who have their need for family planning satisfied with modern methods
3.8 Achieve universal healthcoverage, including financial risk protection, access to quality essential health-care services and access to safe, effective, quality and affordable essential medicines and vaccines for all	3.8.1 Coverage of essential health services (defined as the average coverage of essential services based on tracer interventions that include (…), among the general and the most disadvantaged population) 3.8.2 Proportion of population with large household expenditures on health as a share of total household expenditure or income
3.9 By 2030, substantially reduce the number of deaths and illnesses from hazardous chemicals and air, water and soil pollution and contamination	3.9.1 Mortality rate attributed to household and ambient air pollution 3.9.2 Mortality rate attributed to unsafe water, unsafe sanitation and lack of hygiene (exposure to unsafe Water, Sanitation and Hygiene for All (WASH) services)
3.A Strengthen the implementation of the World Health Organization Framework Convention onTobacco Control in all countries, as appropriate	3.A.1 Age-standardized prevalence of current tobacco use among persons aged 15 years and older
3. B Support the research and development of vaccines and medicines forthecommunicable and non-communicable diseases that primarily affect developing countries, provide access to affordable essential medicines and vaccines, in accordance with the Doha Declarationon the TRIPS Agreement and Public Health (…)	3.B.1 Proportion of the population with access to affordable medicines and vaccines on a sustainable basis 3.B.2 Total net official development assistance to medical research and basic health sectors
3.C Substantially increase health financing and the recruitment, development, training and retention of the health workforce in developing countries, especially in least developed countries and small island developing States	3.C.1 Health worker density and distribution
3.D Strengthen the capacity of all countries, in particular developing countries, for early warning, risk reduction and management of national and global health risks	3.D.1 International Health Regulations (IHR) capacity and health emergency preparedness
**SDG 11: Make cities inclusive, safe, resilient and sustainable**	
11.1 By 2030, ensure access for all to adequate, safe and affordable housing and basic services and upgrade slums	11.1.1 Proportion of urban population living in slums, informal settlements or inadequate housing
11.2 By 2030, provide access to safe, affordable, accessible and sustainable transport systems for all, improving road safety, notably by expanding public transport, with special attention to the needs of those in vulnerable situations, women, children, persons with disabilities and older persons	11.2.1 Proportion of population that has convenient access to public transport, by sex, age and persons with disabilities
11.3 By 2030, enhance inclusive and sustainable urbanization and capacity for participatory, integrated and sustainable human settlement planning and management in all countries	11.3.1 Ratio of land consumption rate to population growth rate 11.3.2 Proportion of cities with a direct participation structure of civil society in urban planning and management that operate regularly and democratically
11.6 By 2030, reduce the adverse per capita environmental impact of cities, including by paying special attention to air quality and municipal and other waste management	11.6.1 Proportion of urban solid waste regularly collected and with adequate final discharge out of total urban solid waste generated, by cities 11.6.2 Annual mean levels of fine particulate matter (e.g. PM2.5 and PM10) in cities (population weighted)
11.7 By 2030, provide universal access to safe, inclusive and accessible, green and public spaces, in particular for women and children, older persons and persons with disabilities	11.7.1 Average share of the built-up area of cities that is open space for public use for all, by sex, age and persons with disabilities 11.7.2 Proportion of persons victim of physical or sexual harassment, by sex, age, disability status and place of occurrence, in the previous 12 months
11. B By 2020, substantially increase the number of cities and human settlements adopting and implementing integrated policies and plans towards inclusion, resource efficiency, mitigation and adaptation to climate change, resilience to disasters, and develop and implement, in line with the Sendai Framework for Disaster Risk Reduction 2015–2030, holistic disaster risk management at all levels	11.b.1 Proportion of local governments that adopt and implement local disaster risk reduction strategies in line with the Sendai Framework for Disaster Risk Reduction 2015–2030 11.b.2 Number of countries with national and local disaster risk reduction strategies
**SDG 2. End hunger, achieve food security and improved nutrition and promote sustainable agriculture**	
2.2 By 2030, end all forms of malnutrition, including achieving, by 2025, the internationally agreed targets on stunting and wasting in children under 5 years of age, and address the nutritional needs of adolescent girls, pregnant and lactating women and older persons	2.2.1 Prevalence of stunting (height for age < −2 standard deviation from the median of the World Health Organization (WHO) Child Growth Standards) among children under 5 years of age 2.2.2 Prevalence of malnutrition (weight for height > + 2 or < − 2 standard deviation from the median of the WHO Child Growth Standards) among children under 5 years of age, by type (wasting and overweight)
**SDG 4. Ensure inclusive and equitable quality education and promote lifelong learning opportunities for all**	
4.A Build and upgrade education facilities that are child, disability and gender sensitive and provide safe, non-violent, inclusive and effective learning environments for all	4.A.1 Proportion of schools with access to: (a) electricity; (b) the Internet for pedagogical purposes; (c) computers for pedagogical purposes; (d) adapted infrastructure and materials for students with disabilities; (e) basic drinking water; (f) single- sex basic sanitation facilities; and (g) basic handwashing facilities (as per the WASH indicator definitions)
**SDG 6: Ensure availability and sustainable management of water and sanitation for all**	
6.1 By 2030, achieve universal and equitable access to safe and affordable drinking water for all	6.1.1 Proportion of population using safely managed drinking water services
6.2 By 2030, achieve access to adequate and equitable sanitation and hygiene for all and end open defecation, paying special attention to the needs of women and girls and those in vulnerable situations	6.2.1 Proportion of population using safely managed sanitation services, including a hand-washing facility with soap and water
6.3 By 2030, improve water quality by reducing pollution, eliminating dumping and minimizing release of hazardous chemicals and materials, halving the proportion of untreated wastewater and substantially increasing recycling and safe reuse globally	6.3.1 Proportion of wastewater safely treated 6.3.2 Proportion of bodies of water with good ambient water quality
**SDG 7. Ensure access to affordable, reliable, sustainable and modern energy for all**	
7.1 By 2030, ensure universal access to affordable, reliable and modern energy services	7.1.1 Proportion of population with access to electricity 7.1.2 Proportion of population with primary reliance on clean fuels and technology
7.2 By 2030, increase substantially the share of renewable energy in the global energy mix	7.2.1 Renewable energy share in the total final energy consumption
**SDG 8. Promote sustained, inclusive and sustainable economic growth, full and productive employment and decent work for all**	
8.8 Protect labour rights and promote safe and secure working environments for all workers, including migrant workers, in particular women migrants, and those in precarious employment	8.8.1 Frequency rates of fatal and non-fatal occupational injuries, by sex and migrant status
**SDG 9: Build resilient infrastructure, promote inclusive and sustainable industrialization and foster innovation**	
9.5 Enhance scientific research, upgrade the technological capabilities of industrial sectors in all countries, in particular developing countries, including, by 2030, encouraging innovation and substantially increasing the number of research and development workers per 1 million people and public and private research and development spending	9.5.1 Research and development expenditure as a proportion of GDP
9.A Facilitate sustainable and resilient infrastructure development in developing countries through enhanced financial, technological and technical support to African countries, least developed countries, landlocked developing countries and small island developing States	9.A.1 Total official international support (official development assistance plus other official flows) to infrastructure
9.C Significantly increase access to information and communications technology and strive to provide universal and affordable access to the Internet in least developed countries by 2020	9.C.1 Proportion of population covered by a mobile network, by technology
**Goal 12. Ensure sustainable consumption and production patterns**	
12.1 Implement the 10-Year Framework of Programmes on Sustainable Consumption and Production Patterns, all countries taking action, with developed countries taking the lead, taking into account the development and capabilities of developing countries	12.1.1 Number of countries with sustainable consumption and production (SCP) national action plans or SCP mainstreamed as a priority or a target into national policies
12.3 By 2030, halve per capita global food waste at the retail and consumer levels and reduce food losses along production and supply chains, including post-harvest losses	12.3.1 Global food loss index
12.4 By 2020, achieve the environmentally sound management of chemicals and all wastes throughout their life cycle, in accordance with agreed international frameworks, and significantly reduce their release to air, water and soil in order to minimize their adverse impacts on human health and the environment	12.4.2 Hazardous waste generated per capita and proportion of hazardous waste treated, by type of treatment
12.5 By 2030, substantially reduce waste generation through prevention, reduction, recycling and reuse	12.5.1 National recycling rate, tons of material recycled
12.B Develop and implement tools to monitor sustainable development impacts for sustainable tourism that creates jobs and promotes local culture and products	12.B.1 Number of sustainable tourism strategies or policies and implemented action plans with agreed monitoring and evaluation tools
12.C Rationalize inefficient fossil-fuel subsidies that encourage wasteful consumption by removing market distortions, in accordance with national circumstances, including by restructuring taxation and phasing out those harmful subsidies, where they exist, to reflect their environmental impacts, taking fully into account the specific needs and conditions of developing countries and minimizing the possible adverse impacts on their development in a manner that protects the poor and the affected communities	12.C.1 Amount of fossil-fuel subsidies per unit of GDP (production and consumption) and as a proportion of total national expenditure on fossil fuels
**Goal 13. Take urgent action to combat climate change and its impacts**	
13.1 Strengthen resilience and adaptive capacity to climate-related hazards and natural disasters in all countries	13.1.1 Number of deaths, missing persons and directly affected persons attributed to disasters per 100,000 population 13.1.3 Proportion of local governments that adopt and implement local disaster risk reduction strategies in line with national disaster risk reduction strategies
13.2 Integrate climate change measures into national policies, strategies and planning	13.2.1 Number of countries that have communicated the establishment or operationalization of an integrated policy/strategy/plan which increases their ability to adapt to the adverse impacts of climate change, and foster climate resilience and low greenhouse gas emissions development in a manner that does not threaten food production (including a national adaptation plan, nationally determined contribution, national communication, biennial update report or other)
***Leaving no one behind: health equity as a crosscutting issue***	
**Goal 1.End poverty in all its forms everywhere**	
1.1 By 2030, eradicate extreme poverty for all people everywhere, currently measured as people living on less than $1.25 a day	1.1.1 Proportion of population below the international poverty line, by sex, age, employment status and geographical location (urban/rural)
1.2 By 2030, reduce at least by half the proportion of men, women and children of all ages living in poverty in all its dimensions according to national definitions	1.2.1 Proportion of population living below the national poverty line, by sex and age
1.3 Implement nationally appropriate social protection systems and measures for all, including floors, and by 2030 achieve substantial coverage of the poor and the vulnerable	1.3.1 Proportion of population covered by social protection floors/systems, by sex, distinguishing children, unemployed persons, older persons, persons with disabilities, pregnant women, newborns, work-injury victims and the poor and the vulnerable
1.4 By 2030, ensure that all men and women, in particular the poor and the vulnerable, have equal rights to economic resources, as well as access to basic services, ownership and control over land and other forms of property, inheritance, natural resources, appropriate new technology and financial services, including microfinance	1.4.1 Proportion of population living in households with access to basic services
**Goal 5. Achieve gender equality and empower all women and girls**	
5.1 End all forms of discrimination against all women and girls everywhere	5.1.1 Whether or not legal frameworks are in place to promote, enforce and monitor equality and non-discrimination on the basis of sex
**SDG 10: Reduce inequalities within and among countries**	
10.3 Ensure equal opportunity and reduce inequalities of outcome, including by eliminating discriminatory laws, policies and practices and promoting appropriate legislation, policies and action in this regard	10.3.1 Proportion of the population reporting having personally felt discriminated against or harassed within the previous 12 months on the basis of a ground of discrimination prohibited under international human rights law
***Policy coherence, governance, stakeholders’ participation, need of high quality information and data***	
**SDG 16. Promote peaceful and inclusive societies for sustainable development, provide access to justice for all and build effective, accountable and inclusive institutions at all levels**	
16.7 Ensure responsive, inclusive, participatory and representative decision-making at all levels	16.7.1 Proportions of positions (by sex, age, persons with disabilities and population groups) in public institutions (national and local legislatures, public service, and judiciary) compared to national distributions 16.7.2 Proportion of population who believe decisionmaking is inclusive and responsive, by sex, age, disability and population group
**SDG 17. Strengthen the means of implementation and revitalize the Global Partnership for Sustainable Development**	
17.14 Enhance policy coherence for sustainable development	17.14.1 Number of countries with mechanisms in place to enhance policy coherence of sustainable development
17.16 Enhance North-South, South-South and triangular regional and international cooperation on and access to science, technology and innovation and enhance knowledge sharing on mutually agreed terms, including through improved coordination among existing mechanisms, in particular at the United Nations level, and through a global technology facilitation mechanism	17.6.1 Number of science and/or technology cooperation agreements and programmes between countries, by type of cooperation
17.18 By 2020, enhance capacity-building support to developing countries, including for least developed countries and small island developing States, to increase significantly the availability of high-quality, timely and reliable data disaggregated by income, gender, age, race, ethnicity, migratory status, disability, geographic location and other characteristics relevant in national contexts	17.18.1 Proportion of sustainable development indicators produced at the national level with full disaggregation when relevant to the target, in accordance with the Fundamental Principles of Official Statistics

The SDGs are an indivisible and interdependent set of goals. Further, the discussion on sustainable development at Rio + 20 introduced the notion of health co-benefits [22], upgrading the profile of health within the sustainable development agenda. Health outcomes are good mobilizers of policies from other sectors that may be unpopular such as traffic restrictions or speed limits within cities. As mentioned in the theoretical framework, cities that work towards decreasing air and noise pollution and increasing physical exercise or green open spaces can dramatically reduce the incidence, morbidity, mortality and associated costs of a wide range of diseases, from heart disease and stroke, to cancer and mental health issues.

A critique made of the 2030 Agenda is that action on some SDGs could have reinforcing but also counteracting or cancelling effects on planetary or human health. For example, achievement of food security is fundamentally dependent on increasing production, improving quality and ensuring access. At the same time, agricultural production is a major source of environmental impact, including climate change [23]. Implicit in the SDG framework, is to consider interactions between different goals and sectors in ways that promote policy coherence, and the need to continuously monitor and evaluate progress through a set of largely agreed targets and indicators. HiAP proposes a systemic and multisectorial approach within a Planetary Health vision, which takes into account human health as well as the planet’s health [24] to help articulate these SDGs interactions.

Intersectoral work is usually hard to fund, since resources fall across several institution’s budgets. The 2030 Agenda, signed by national presidents, and usually under their mandate, could support mechanisms for partnership activities and joint budgeting, particularly at a subnational or local level, where implementation of comprehensive public health policies in many resource-constrained settings has been strong. For example, early childhood development programmes have demonstrated significant long-term health and socioeconomic advantages, while in many LMICs, water and sanitation policies have developed together with the health sector [25]. In places that heavily rely on external aid, HiAP can be an extraordinary asset to show donors predictable, coherent and sustainable results (see SDG 17 indicators in Table 1).

#### 2) Health equity: a cross cutting issue for the SDGs

The WHO Commission on Social Determinants of Health states that social inequalities in health arise from inequalities in the conditions of daily life, with the inequities in power, money and resources being the main drivers. These social and economic inequalities underpin the determinants of health or the range of interacting factors that shape health and wellbeing [26]. Cities are faced with dramatic demographic and epidemiological transitions, and produce great health inequalities [27], for instance, in the form of segregation by social class, gender, age or ethnicity within or among city’s neighborhoods, and an increase to 828 million people living in slums worldwide [4].

HiAP gained momentum in the last decade through the intensive debate on action for social determinants of health, and because of this, a particular focus has been on the equity dimension. Health equity for those more vulnerable and most often exposed to risks, such as children, older people, women, people with disabilities, and the poor is essential to ensure health gains are distributed equally. In parallel, equity is both a crosscutting issue for the 2030 Agenda, whose general motive is “leave no one behind”, but also specific objectives (SDGs 2, 5 and 10). For gender equality, the overwhelmingly positive interactions with other goals suggests that actions for improved gender equality can be an important lever overall.

Several biases are commonly presented in urban and transport planning, for example, in the mobility area where most interventions are based on the necessities and perspectives of those healthy, wealthy and male [28, 29]. Participatory processes required to identify inclusive priorities across vulnerable subpopulations (women, elderly, those living with disabilities, etc.) will strongly impact the achievement of SDG5 and SDG10.

#### 3) Policy coherence, governance, stakeholders’ participation, need of high quality information and data

The SDGs, unlike the Millennium Development Goals (MDGs), address political governance and challenge governments and partners to be more political, systemic and holistic. SDG 17 calls for stronger commitment to partnership and cooperation by establishing policy coherence and an enabling environment for sustainable development at all levels and by all actors. This illustrates the possibility of the SDGs to provide a platform to encourage further research and understanding of effective governance, funding and partnership structures to develop sustainable solutions towards Health for All. Institutional and technical core capacities can be strengthened across countries through international efforts. The long-term and sustained efforts required by HiAP usually do not match those of shorter political cycles. The SDGs proposed 2030 as the horizon to achieve those goals, thus, providing a 15-year cycle (10 years remaining at the time of this publication) to work on key solutions for complex sustainability problems.

HiAP also helps navigate this policy-making process by, for example, encouraging the involvement of all major stakeholders in urban health or promoting community-citizenship active participation (see SDG 16 about participatory institutions) in health impact assessments.

The success or failure of the SDGs will depend, in large part, upon effective monitoring. Well-crafted indicators and high quality data will give governments, businesses, academia, and civil society the information they need to target resources, policies, and programs. The current set of “official” indicators were proposed by the Statistical Commission of ECOSOC in 2017, and developed by the Inter-Agency and Expert Group on SDG indicators (IAEG-SDGs) through an open and transparent process involving many stakeholders. There are currently more than 200 indicators, although the indicators and monitoring continue to evolve [30]. UN annual reports provide track of progress on the objectives over time in critical areas, favorable trends, but also areas that need urgent collective attention both globally and by region [31]. Other stakeholders, mostly at academic sectors, are modelling and validating composite measures, e.g. SDG Index, able to synthetically compare results across time and geographically [32].

### HiAP implementation: lessons learnt from health impact assessments on urban health

HiAP implementation could involve: laws, regulations or agreements such as the international Framework Convention on Tobacco Control or National Health Care Acts that consider health impacts of other policies; structures such as inter-ministerial committees, expert commissions or support units within ministries of health or public health institutes; and processes such as consultations, strategies or reporting systems such as strengthened public health surveillance [2].

Within a HiAP approach, HIA is one of the main tools for urban decision-makers to apply a “health lens” to fully assess the risks and opportunities posed by policies and programs and measure the health effects. The WHO defines HIAs: “a means of assessing the health impacts of policies, plans and projects in diverse economic sectors using quantitative, qualitative and participatory techniques”. The last decade has seen a significant growth in the use of HIAs, and some countries (Wales, Australia, Thailand and Brazil) are formalizing their use in the decision-making process [33].

Nieuwenhuijsen et al. [34] define integrated full-chain HIA modelling as those assessments that analyze from determinants, through pathways, to health impacts, considering multiple exposures and complexities, interdependencies and uncertainties of the real world. HIAs are tagged as participatory when they entail stakeholders and citizens’ visions and necessities, aimed at successful implementation and policy utility maximization. The most common HIAs in urban contexts are qualitative, aiming only to identify the range of the health determinants associated with a policy or intervention, and the direction of its impacts (risk versus benefit). HIAs can also include a quantitative assessment by comparing current burden of disease (e.g. cases of disease, injuries, deaths, or disability adjusted life years [DALY]) estimation, with the health impacts of a future change associated with a proposed intervention or policy [34]. This quantitative estimate of the expected health impact can be applied to different policy scenarios, helping stakeholders and policy makers to take decisions based on health evidence. Quantitative HIAs can use international exposure recommendation values, when available, as goals to be achieved by different policy scenarios. Existing HIA evaluation tools developed to be used in the field of Urban Health include: the Health Economic Assessment Tool (HEAT) for walking and cycling [35], the Integrated Transport and Health Impact Modeling (ITHIM) [36], the Transportation, Air pollution and Physical Activities (TAPAS) [37], the Urban and Transport Planning Health Impact Assessment (UTOPHIA) [38], or the Blue Active Tool [15]. Table 3 captures a range of first-hand HIAs by coauthors ranging from the city of Barcelona, where the group has extensive experience, to other cities in Europe, Latin America and Africa.

Table 3. Examples of HIAs conducted by coauthors.

Based on the experience gathered through these HIAs, we discuss the particular contexts for the three main HiAP enablers described above (supportive contexts, resources and skills to assess health impacts of other policies, and information and data availability), focusing on main barriers and bottlenecks but also opportunities to achieve the SDGs.

**Table 3:** Examples of HIAs conducted by coauthors

Location	Methodology/ Tool	Exposure	Outcome	Related Policy	Main opportunities	Main barriers	Author
Barcelona	Blue Active Tool	Physical activity	Mortality, morbidity, DALYs, health economic values	Riverside regeneration	First impact assessment on this topic in this city	Data quality & availability No stakeholder participation	Vert et al, 2019 [15]
Barcelona	UTOPHIA	Transport-PA Air pollution (PM2.5) Noise (Lday 16h) Green space (%GS) Heat (daily mean Temperature)	Mortality	Local urban and transport planning policies	Holisitic approach in estimating the mortality burden associated with the multiple urban and transport planning related exposures Detailed exposure data on same spatial scale Quantification of expected impact, direction and magnitude of expected health effects	Uncertainty in causal inferences Mortality is ‘tip of the iceberg’, morbidity was not considered ‘Double-counting’ of cases (correlation of exposures) Risk of exposure and outcome misclassification Quantitative HIA cannot capture intrinsic motivations for behaviour change	Mueller et al 2017a [38]
Barcelona	UTOPHIA	Transport-PA Air pollution (PM2.5) Noise (Lday 16h) Green space (%GS) Heat (daily mean Temperature)	Morbidity DALYs	Local urban and transport planning policies	Holistic approach in estimating the mortality burden associated with multiple exposures Detailed exposure data on same spatial scale Quantification of expected impact, direction and magnitude of expected health effects	Uncertainty in causal inferences DALY estimations & scaling from national to local level uncertainty Risk of exposure and outcome misclassification	Mueller et al. 2017b [39]
Port Louis	Health Risk Assessment	Physical activity, travel mode, heat, air pollution	Mortality	Light Metro Express Rail	Smaller city: faster process, primary data collection feasible, less costly Collaboration of all relevant ministries and sectors Easily replicable model Scalability if time and financial resources exist	Data quality & availability, gap between policy concerns and citizens needs Lack of interest by local media	Thondoo et al. Unpublished
Argentina: Rosario; Bolivia: El Alto; Brazil: Sao Paulo; Chile: Santiago de Chile; Colombia: Bogota, Cali and Medellin; Ecuador: Cuenca and Quito; Guatemala: Guatemala City; Mexico: Guadalajara, Mexico City and Puebla; Panama: Panama City; Peru: Lima	Blue Active Tool	Physical activity	Mortality, morbidity, DALYs, and mortality related economic values	Open streets	First multinational impact assessment on Open Streets Near collaboration with stakeholders Using the results for advocacy	Data availability and quality Lack of harmonize data between countries and cities Lack of collaboration between health sector and urban /transport planning	Velazquez-Cortez D, et al, unpublished
Mexico City Bogota Istanbul Brisbane Paris	TAPAS tool	Physical activity, air pollution, traffic incidents	Mortality and mortality related economic values	Bus Rapid Transit	First impact assessment Policy popularity	Data availability and quality Lack of harmonized data between countries and cities Lack of collaboration between health sector and urban /transport planning	Rojas-Rueda D et al, unpublished
Barcelona	TAPAS tool	Physical activity, air pollution, traffic incidents	Mortality and mortality related economic values	Tram expansion	Stakeholder collaboration Use analysis data to decision making process and political debate	Data quality & availability Lack of interest from different political parties at city council	Rojas-Rueda D et al, [40]
Barcelona	TAPAS tool	Physical activity, air pollution, traffic incidents	Mortality	Bike sharing systems	First assessment Results helped to strength collaboration between local stakeholders and researchers	Data quality & availability No stakeholder participation	Rojas-Rueda D et al, 2011 [37]
Barcelona	TAPAS tool	Physical activity, air pollution, traffic incidents	Mortality	Active transportation (walking and cycling) and public transport scenarios	First assessment Results helped to strength collaboration between local stakeholders and researchers	Data quality & availability No stakeholder participation	Rojas-Rueda D et al, 2012 [41]
Barcelona	TAPAS tool	Physical activity, air pollution, traffic incidents	Mortality, morbidity and DALYs	Active transportation (walking and cycling) and public transport scenarios	Provide in deep analysis of transport related impact at local level Results helped stakeholders to understand transport and health pathways	Data quality & availability No stakeholder participation	Rojas-Rueda D et al, 2013 [42]
Barcelona Brussels Hamburg Lille Lyon Madrid Milan Paris Seville Toulouse Valencia Warsaw	TAPAS tool	Physical activity, air pollution, traffic incidents	Mortality	Bike sharing systems	First multinational assessment Results helped to strength regional advocacy on bike sharing systems	Data quality & availability Lack of harmonized data between countries and cities No stakeholder participation	Otero I et al, 2018 [43]
Barcelona Basel Copenhagen Paris Prague Warsaw	TAPAS tool	Physical activity, air pollution, traffic incidents	Mortality	Walking and cycling	Policy comparative between multiple cities and countries, helping to understand the implications of similar polices in different context and locations.	Data quality & availability Lack of harmonized data between countries and cities No stakeholder participation	Rojas-Rueda D et al, 2016 [44]
Morocco	Quantitative Health Impact Assessment	Air pollution, water and sanitation	Mortality	SDGs 3, 6 and 11 implementation	SDG context & stakeholder support	Data quality & availability	Rojas-Rueda D et al, 2018 [45]
Maputo Cochabamba	Evaluating feasibility, pilot study	NA	NA	NA	Fast urban growing of the cities and low development; Possibilities for policy and intervention assessment; Intersectorial approaches; Establishment of new communication pathways between local authorities.	Low regulations; Low continuity in the policies; Low cooperation between stakeholders and poor long-term engagement; Low understanding of HIA Research Low capacity to collect routinely data; Low comparability between data collected.	Gascon et al. 2016 [46]
Antwerp Barcelona London Örebro Rome Zurich	Health impact assessment of cycling network expansions in European cities	Transport mode physical activity air pollution (PM2.5) fatal traffic accidents	Mortality	Cycling infrastructure policies	First study evaluating the potential associations between cycling network length, mode share and associated health impacts across European cities Standardized data extraction methods	Detailed data availability Cross-sectional study, no causality can be implied Detailed data availability, assumptions on causal inferences Impacts estimated only for active travelers, societal benefits ignored	Mueller et al, 2018 [48]
Bradford	UTHOPIA	Transport mode physical activity air pollution (PM2.5) noise (Lden) green space (%GS) Index Multiple Deprivation Ethnicity	Mortality	Local urban and transport planning policies	Holisitic approach in estimating the mortality burden associated with the multiple urban and transport planning related exposures Distribution of mortality burden by SES variables	Assumptions on causal inferences were evidence is lacking Risk of exposure and outcome misclassification Different strengths of evidence for exposures with mortality Quantitative HIA cannot captures intrinsic motivations for behavior change	Mueller et al, 2018 [49]
Barcelona		Transport-related physical activity (PA), (air pollution (NO2), road traffic noise, green space, urban heat island (UHI) effect	Premature mortality, changes in life expectancy and economic impacts	Local planning (Superblock model)	Robust overall estimation, based on best epidemiological evidence according to the current research. Multiple urban and transport planning related exposures were considered holistically and where uncertainty on causal inferences existed, assumptions were defined with caution and impacts were estimated conservatively. Research conducted by academia in consortium with public health and urban ecology local agencies	Non-fatal impacts such as the expected reduction in chronic disease, improvements in quality of life, social cohesion and mental health have not been quantified. Simultaneous improvement of the suburban commuter network is needed. Gentrification is a possible risk. Undesired relocation of car/motorcycle traffic (to potentially already deprived areas) outside the Superblocks needs to be considered and avoided	Muller et al, 2019 [47]

#### a) A political, legal and health governance supportive context

The primary condition necessary to conduct a HiAP approach is a supportive political and legal context. Within the examples provided in Table 3, the ongoing collaboration between ISGlobal and local authorities has been evolving over many years. Barcelona City Council and other municipal and metropolitan area officers have been sensitized about the links among health and urban and transport planning, resulting in their frequent request for academia to asses or incorporate the best evidence in these policy arenas. In this cooperative exchange, researchers are provided access to local data and funding to produce HIAs in the metropolitan area.

Mozambique, Bolivia and Morocco provide examples of ISGlobal’s long-term strategic alliances, focusing on infectious and neglected diseases and other global health issues for the past several decades. When the former ISGlobal merged with the environmental health research center (CREAL), the rationale was to extend the existing research portfolio (including urban health) in these countries, in consortia with our partners. In Mozambique, there was no knowledge of HIA as a tool, nor was there the capacity to work among different sectors. Although the transport and urban planning authorities acknowledged the links with health, working across sectors was not seen as relevant due to the overlap in competencies and the lack of impact in meeting their own specific sector’s objectives [39]. In Bolivia, some incipient interest on the use of health to promote non-health policies was shown [39]. In the collaboration between ISGlobal and the government of Morocco [40], a burden of disease approach was conducted to compare current levels with 2030 targets on air pollution and water, sanitation and hygiene (WASH) at national level, using the SDG framework. This latter exercise incorporated a HiAP approach integrating multiple stakeholders and authorities from different sectors (such as ministers of interior, infrastructure, water, environment and health) to provide a snapshot of the current SDG situation in Morocco for air pollution and WASH, and listed the evidence-based effective interventions from a health perspective that needed to be implemented in each sector to achieve the SDGs by 2030.

In Mauritius [Thondoo, unpublished], the overall aim of the HIA was to assess the health impacts of urban and transport planning on residents of the capital city Port Louis. Stakeholders from the Ministry of Health as well as the Ministry of Public Infrastructure and Land Transport and technical bodies (for example: statistical departments, climate meteorological stations), non-governmental and multilateral organisations and resident groups participated in the scoping phase. Stakeholders were aware of the use of HIAs in other settings, but had never conducted or supervised one at the local level. The country has no legislation on HIA; although health is sometimes (and not systematically) assessed as part as wider project-driven Environmental Impact Assessments. Interviews and focus groups were used as part of the initial screening process of the HIA and contributed to identifying the local needs, framing the issue and selecting indicators for the HIA that were then contrasted and assembled during focus group discussions to co-create a final HIA design. Stakeholders reported that there was a crucial need to build cross-sectorial platforms and opportunities to discuss health impacts of non-health sectors such as urban transport planning.

#### b) Assessing health impacts of policies in other sectors, including social determinants of health

The information generated by many of these HIAs has been key to support local policies that promote cycling, walking, public, zero and low-emitting modes of transport, and the provision of urban greening or healthy public open spaces. Examples presented in Table 3 show the barriers and opportunities related to quantitative HIAs, that have informed current Urban Mobility, public transportation (tram expansion), green infrastructure and biodiversity plans in Barcelona, the Bus Rapid Transit in six cities around the world, the popular urban initiative on Open Streets in fifteen Latin American cities, or Light Metro Express Rail in Port Louis (Mauritius). In Mauritius, the specific focus was on assessing the urban health-related SDG target indicator 11.2.1, related to access to public transport by sex, age, and persons with disabilities. In view of different public transport measures currently being implemented on the island, stakeholders considered this indicator relevant to assess. In another example, a holistic approach was used to evaluate the health impacts of the multiple urban and transport planning related exposures linked to the Superblock, an urban model intervention in Barcelona [41], incorporating the best epidemiological evidence on the health impacts resulting from the reduction in private motorized transport and changes towards more active and sustainable mobility, increases green and public open space, and mitigation of climate change impact.

Economic impacts of different policy scenarios, often part of HIAs, are useful in allowing decision makers to target their actions so they can make cost effective decisions (e.g. annual costs that could be avoided under compliance with exposure recommendations). Several of the HIAs estimated direct health costs or mortality economic values (based on value of statistical life). For example, Mueller et al. [42] estimated that 2904 deaths or 52,000 DALYs (13% of all annual DALYs) could be prevented annually if Barcelona complied with international recommendations for five main environmental exposures, and that an average resident could live almost one additional year. This would result in 9.3 billion euros of annual savings (from prevented deaths), plus 20 million euros annually from associated morbidity. Cost-benefit assessments can also consider different scenarios for specific interventions. For example, expansion of cycling networks at different rates (i.e. from 10% to an “all streets” scenario) could avoid up to 1000 premature deaths annually in several European cities, mainly due to benefits from increases in physical exercise rates, and even when taking into account increased exposure to air pollution and traffic accidents by cyclists [43].

Providing results by socio-economic status is key to incorporate health equity issues in urban policies and to identify the most vulnerable populations that urgently need policy action. In the examples presented, only in the case of Bradford, UK, did authors offer results stratified by deprivation status (using an index that considers seven domains such as income, employment, education, health, crime, barriers to housing and services and living environment), and ethnicity [44]. Including an equity lens in HIAs is critical for a systemic assessment and to improving health for all and ensuring that ‘no one is left behind.’

#### c) The quality of the information on health exposures and outcomes

In several of our examples, a variety of exposures were included: travelling modes, road-traffic injuries, physical activity (PA), air pollution, noise, heat, access to green spaces or access to WASH. Sources of information for these exposures varied greatly, from national or local health surveys, meteorological records, local air pollution or noise data, to sophisticated estimations such as those of the European Study of Cohorts for Air Pollution Effects Land Use Regression (ESCAPE LUR) [45], or satellite images to calculate the NDVI index, a way to estimate exposure to greenness within cities [46]. Using different quantitative methods and tools, authors were able to estimate exposures at census (small area) level [38, 42]. In several of the HIAs conducted, current exposures were compared to recommended exposures using international guidelines such as WHO weekly recommendations for physical activity [47], annual mean PM2.5 exposure concentrations (below 10 μg/m3) for air pollution [48], daytime outdoor noise levels (below 55 dB) for noise pollution [49] or access to green spaces (living within a 300 m linear distance of a green space greater than 0.5 ha) [46, 50]. In cases where no official international guidelines have been established, a cut-off point can be used based on the scientific evidence and knowledge accumulated on the dose-response relationship (e.g. heat exposure in cities).

In terms of health outcomes, a key source of information throughout the different examples is the Global Burden of Disease study [51]. For example, Mueller et al. [42] used national estimates provided by this study to calculate city-level burden of disease figures, by scaling those to the city’s population size, age and sex structures. Other sources of information regarding health outcomes rely on national statistics capacity, including national health surveys, national death registries, hospital, or traffic records.

Data availability and quality is one of the main bottlenecks in poor resources settings. For example, Gascon et al. [39] conducted a scoping study based on interviews with different key informants, including National Institutes of Statistics, local administrations, academia, NGOs or development agents, to evaluate the availability of data to conduct quantitative HIA in Maputo (Mozambique) and Cochabamba (Bolivia). Data gaps were extraordinary in the first, where there was no appropriate data on mortality, road traffic accidents, nor physical activity for the general population. Conducting a quantitative HIA was not feasible for such contexts. In Bolivia, data on traffic injuries and mortality data was available while more sophisticated information (traffic flows, mobility surveys and transport modal shares) would be available soon. In Mauritius, data related to air pollution was quite scarce due to limited numbers of monitoring stations (only 2 on the whole island) and no roadside monitoring, impeding the possibility of conducting PM10 exposure-outcome spatial analysis. Data for daily measures of heat (temperature) is collected by a parastatal-led climate station, and disclosable for research purpose but at very high cost. As in many developing countries, Mauritius does not conduct travel surveys, therefore no data exists on transport modes, lengths and speed, which makes it difficult to assess exposures during commute. Data on physical activity is also not available or collected.

## Discussion

In this article we presented a conceptual framework linking SDGs and urban health to demonstrate that a HiAP approach resonates with “health” as a determinant, outcome and indicator of sustainable development. We found at least 48 SDG targets relevant to urban health, corresponding to 15 SDGs, while 4 important aspects contained in our proposed theoretical framework, were not present in the SDGs (physical activity, noise, quality of life or social capital). Other assessments have also included relevant health-related targets across several SDGs other than SDG3 [51–53]. The Global Burden of Disease study [51] highlights that health crosscuts 10 out of the remaining 16 goals (in addition to SDG3), shapes 28 health-related targets and is present in 47 health-related indicators. For example, health is a precondition of sustainable cities (SDG 11), through access to decent housing, clean air and water, nutritious food, safe transport and mobility, opportunities for physical activity, and protection from injury risks and toxic pollutants, among others.

Additional arguments for why a HiAP rationale should be present within this conceptual framework included: 1) the importance of intersectoral work, 2) health equity as a cross-cutting issue, and 3) bringing attention to policy coherence, health governance, stakeholders’ participation, and the need for high quality information and data. To bring HiAP into practice, opportunities and barriers of performing HIAs and informing policies related to urban planning, transportation and other local interventions have been discussed. The following points discuss these main findings.

### HiAP is a suitable tool for achieving urban health related SDGs

The HiAP approach is key for local decision-making processes that recognize urban policies as key public health interventions aimed at achieving SDG targets. The increasing push towards more effective forms of governance and the systemic nature of public policy in general has also led to a growing interest in HiAP as an innovative way to address health challenges through collaboration among different state or city departments.

The first wave of countries implementing HiAP into their national public policies included countries with sophisticated legislative and organizational models such as Finland or Australia. The South Australian experience deserves closer attention as it stands out for the relatively early adoption of HiAP, which has survived through political transitions and changes of governments. Baum and others [54] assessed whether differences in population health outcomes can be attributed to HiAP being implemented in South Australia They concluded that HiAP has facilitated improved population health in this context through: multiple government departments working together, public servants’ appreciation of how their sectors impact on health, and as an incentive in avoiding health promotion strategies purely based on individual life-style changes. However, the broader social determinants of health and its underpinning factors dictating the distribution of power, money and resources have not been fully addressed by HiAP. Interestingly, many of these pioneer examples, both in developed and middle-income countries, are around transport, urban planning and local investment decisions (e.g. Healthy Neighborhoods in Quito, Ecuador, or the Greater Christchurch Urban Development Strategy, in Canterbury, New Zealand) [33].

Among HiAP implementation challenges is public institutions working in silos (for example environmental and health issues almost invariably fall under different departments), with different mandates, budgets, accountability mechanisms, timing and organizational cultures, plus a tendency towards short-term market-oriented approaches to policy-making, and a lack of monitoring progress and evaluating impacts [55]. The 2030 Agenda could help catalyze momentum to overcome these limitations by putting the focus on intersectoral work, health equity, and bringing resources, improbable partnerships, political will and commitment to the table. For example, Buss et al. [56] analyzed both the regional Plan of Action on HiAP for the Americas approved in 2014 (PAHO), and the implementation of the 2030 Agenda, highlighting the benefits of positioning health in other sectors policies in the wider development agenda. On the other hand, the SDG framework is somewhat limited in capturing every aspect of urban health (see Fig. 1 ). Researchers, public health practitioners and policy-making actors should work to incorporate those elements.

### Health impact assessments are a practical tool to design intersectoral interventions

HIAs can harness potential for SDG implementation, serving as an important tool to monitor or evaluate policies in other sectors by providing empirical evidence. The establishment of specific legal frameworks for HIAs could, in theory, ensure their incorporation in key processes that inform public policies or in procurement procedures. Yet, a major barrier to the advancement of the field remains the scarcity of research and lack of HIA legislation and use in low and middle-income countries [57–59]. A recent systematic literature review found that countries with some type of legal framework for HIAs are also those that have published several peer-reviewed assessments [60]. HIAs are indeed recognized formally as important tools to consider adverse health impacts of changing environments in various developed countries both in Europe and North America [57], and are actively promoted by health agencies in Finland, New Zealand, Switzerland, Germany and the UK.

The SDG agenda could also potentially foster funding mechanisms and international partnerships that allow for knowledge sharing and capacity building, overcoming current barriers of excessively simplified tools, inadequate policy guidelines, weak technical skills, absence of solid environmental baseline databases and lack of scientific collaboration [61]. An example is the UN Sustainable Development Solutions Network (SDSN) that mobilizes global scientific and technological expertise to promote practical solutions for sustainable development, including SDGs implementation and the Paris Climate Agreement. By working closely with UN agencies, multilateral financing institutions, the private sector, and civil society, this network is able to leverage funding from diverse and untraditional sources [52].

Through a series of case studies, the usefulness of HIAs to analyze the impact of interventions and policies on a variety of urban health topics has been explored. Full-chain HIAs, sensitive to policy dynamics and settings, for example, go through several rounds of implementation/re-evaluation or feedback, but these are seldom implemented. Haigh et al. states that in Australia policy makers believe that HIAs are ‘expensive and time-consuming’ [62], when evidence actually shows that the benefits derived from HIAs outweigh the cost of undertaking them [63]. For example, in Nigeria a complete health baseline information was one of the critical barriers found on the first steps of a HIA, with new cross-sectional studies compensating for the lack of reliable data [64].

HIAs rely on policy makers being able and willing to invest in or collaborate with institutions in order to complement existing datasets with newly conducted surveys and studies. Policy makers also need to account for time to engage in processes like HIA and report their engagement transparently. This engagement process is crucial and demands rigorous commitment from participating stakeholders (being consistent and engaging at different stages) and HIA practitioners (using scientific methods for sampling and interviewing stakeholders and reporting such methods with accuracy). From the examples provided in this article, those in Barcelona correspond to a long-term trust and collaboration between ISGlobal and local authorities. The ISGlobal experience has been that an initial HIA, even if initiated by one partner on a specific topic invariably leads to the building of relationships of trust and future collaborations among many participants. For instance, the Barcelona quantitative HIA of five urban exposures presented the City Council with (at the time) a unique and comprehensive way to understand how the urban environment could impact health. The study received media coverage and led to further engagement of ISGlobal scientists with city technical and political officers to translate the findings. Notably, it provided valuable and visualizable data to both advocate for and initiate urban planning interventions taking into account health. Over time, this has expanded into a broader set of collaborations that have included policy-planning directives such as the new Regional Mobility Plan 2020–2025 approved in 2019 or HIAs for specific interventions such as the Superblocks [41]. The Superblock HIA is a significant step in demonstrating clearly how changes in the urban model can be explicitly seen as public health interventions. It also illustrates the need to include indicators and monitoring as part of such interventions in order to demonstrate effectiveness and reach established targets (something not included as part of the initial Superblocks program). In Mozambique [39], the HIA exploration led to greater awareness of the links between environment and health in a context still very oriented towards infectious diseases. It created interest in environmental health in urban contexts and has led to the development of several proposals that would further research along these lines.

Because HIAs engage multiple actors across diverse sectors, they can illustrate clearly how HiAP can be an attractive and effective framework for systemic thinking about health. A key asset of a full-chain HIA process is precisely the involvement of citizens and members of different communities whose voices are not usually heard [33], and this is also a fundamental requirement to sustainable development and the 2030 Agenda (see SDG 16.7).

The lack of high quality and sensitive information, particularly in LMIC/poor-resources settings, is one of the main barriers to undertaking full-chain HIAs to fully implement and monitor progress towards the SDGs. The challenges related to scarcity of data as highlighted by authors are consistent with barriers reported elsewhere. This was the case in Maputo, Mozambique [39], but also HIAs conducted in Peru [65], Brazil [66], Iran [67], and Turkey [68], which also reported that using datasets of weak quality makes it difficult to conduct HIA. In addition, resources and skills have to be identified to adapt HIA approaches to each context by making assumptions during modelling, by using non-local dose-response functions and complementing local data with disease data from other settings. For outcome data, the “Global Burden of Disease” is a valuable source of information, particularly for LMICs, since it is the most comprehensive worldwide observational epidemiological study to date [45]. In recent years, the study results have adapted to provide measures for 33 health-related SDG indicators and introduce an overall health-related SDG index for 188 countries, from 1990 to 2015, thus also providing temporal trends. However, GBD estimates only exist at the national and regional level, but not at the local/ city-level, thus bringing too much uncertainty when measuring impact at a small area scale.

The 2030 Agenda calls for a data revolution that moves away from traditional statistical methods. Some examples of data being used in novel ways to assess the implementation of the SDGs include Big Data and social media data, such as mobile phone data and satellite data. Increasing numbers of studies use real-time google traffic data to assess air pollution, since traffic-related black carbon levels can be associated with congestion colors displayed on crowd-sourced traffic maps [69]. The use of this inexpensive tool holds great promise in air pollution modeling worldwide, including places where national statistics or field monitoring do not meet international standards.

Lastly, a key aspect of urban health SDG implementation is ensuring that the commitment to leave no one behind is translated into effective action. This requires an accurate understanding of target populations, their needs and circumstances. Available information must be disaggregated according to the main axes of inequalities such as social class, gender, age or ethnicity/migration. Appropriate tools and metrics that should be widely available include the Urban Health Index, which provides information about health inequalities in small areas within cities or the Urban Health Equity Assessment and Response Tool [70], that both measures and takes action to tackle inequalities. However, the information needed in order to measure socioeconomic inequalities efficiently (exposure or outcome data by different social groups at the small area level or, ideally, geo-referenced) is usually not available, particularly in lower resource settings. Few or none of the current SDG indicators, for example, are able to shed light on the particular situations of migrants, refugees, the elderly, people with disabilities, minorities and indigenous peoples. Thus, the 2030 Agenda should bring significant efforts in the coming years needed to strengthen data collection and countries’ capacity to guarantee equity.

The following is a summary of final recommendations for different stakeholders based on the main key findings and discussion:

- SDGs provide a holistic and integrated framework to address health-related sustainable development challenges. National, regional and local actors should work on SDGs implementation strategies that break administrative silos, bring all public and non-public stakeholders around the table and define plans, budgets and coordination mechanisms. A HiAP approach can help to guide this multi-sector and multi-actor strategy.
- In the case of urban policies, adapted tools should be considered. HIAs, in particular, are an effective way to implement the HiAP approach. This implies building resources and skills within implementation structures, planning for effective engagement strategies for key stakeholders, collecting and making available data and defining evidence-based monitoring and evaluation mechanisms from a health perspective.
- Public institutions and private companies can collaborate in smart ways to collect and manage information systems that define policy design and implementation. General databases such as the GBD can be used as a source to be adapted.
- All layers of health policy design and implementation (from information systems to plans and budgets) must be able to disaggregate exposure and health outcomes by social and income or vulnerable groups in order to guarantee equitable interventions.
- Based on ISGlobal’s experience in Barcelona and within international consortia, long-term trusted collaborations between academia and local policy decision makers, together with a citizen participatory approach add value to the design, implementation and evaluation of urban policies that take into account health.

## Conclusions

HiAP, and applying tools such as HIA, can be used in cities worldwide, including those of less developed regions or countries, to achieve urban health related SDGs in the 2030 Agenda. Data availability, taking into account equity issues, strengthening the communication between experts and citizens, interdisciplinary and interagency collaboration and the involvement of all major stakeholders are crucial elements in a HiAP approach for SDG implementation.
